# Effects of the Remaining and/or Spontaneously Regenerated Facial Axons After Hypoglossal–Facial Nerve Neurorrhaphy for Facial Paralysis

**DOI:** 10.3389/fneur.2020.00413

**Published:** 2020-05-29

**Authors:** Yuan Zhuang, Miao Ling, Zhen Li, Dezhi Li, Hong Wan, Michael Schumacher, Song Liu

**Affiliations:** ^1^Department of Injury and Repair, Beijing Neurosurgical Institute, Beijing, China; ^2^Beijing Key Laboratory of Central Nervous System Injury, Beijing Neurosurgical Institute, Capital Medical University, Beijing, China; ^3^Department of Neurosurgery, Beijing Tiantan Hospital Affiliated to Capital Medical University, Beijing, China; ^4^U1195, INSERM et Universite Paris-Sud, Le Kremlin-Bicêtre, France

**Keywords:** facial nerve injury, hypoglossal nerve, innervation, neurorrhaphy, nerve regeneration

## Abstract

**Background:** The incidence of incomplete facial paralysis is now relatively higher in clinical practice, and surgical intervention is still desirable for patients with significant facial paralysis. However, the importance and usefulness of the remaining and/or spontaneously regenerated facial axons for regaining facial function when using hypoglossal–facial nerve (HN-FN) neurorrhaphy or other nerve-transferring methods to treat facial paralysis remain controversial.

**Objective:** We designed a rat FN injury model with preservation of the anatomical structure followed by HN-FN side-to-side neurorrhaphy to investigate the effects of the remaining and/or spontaneously regenerated FN axons on restoration of facial function.

**Methods:** After the evident return of facial function in 3 months following FN injury and HN-FN side-to-side neurorrhaphy, the FN was cross-sectioned again according to different ratios (0, 30, 70, and 100%) at the site rostral to the initial FN injury to retain, partially abolish, or completely abolish the spontaneously regenerated FN axons that had successfully reinnervated the paralyzed facial muscles. Then, FN function was assessed using clinical evaluation methods and electrophysiological examinations, as well as retrograde labeling and axonal counting assessments of the reconstructed nerve pathways.

**Results:** The evaluations show that the remaining facial axons not only influenced the extent of regained function, such as facial symmetry, eye blinking activity, and vibrissae motion, but also had an impact on regeneration and innervation of hypoglossal motoneurons.

**Conclusion:** Participation of remaining or spontaneously regenerated facial axons plays an important role in innervating paralyzed facial muscles by both facial and hypoglossal motoneurons, thus, reestablishing facial function.

## Introduction

Facial nerve (FN) injury frequently results from cranial trauma, tumor, inflammation, or surgery at the cerebellopontine angle area, inducing either complete or incomplete permanent or transitory facial paralysis. According to the extent of injury, FN injury can be divided into two principal types: preserved or interrupted FN anatomical structure.

Preservation or reestablishment of the anatomical structure of injured FNs has received much attention in neurosurgery in the context of surgery at the cerebellopontine angle area, which leads to the conservation of the remaining and/or possible spontaneous regeneration of injured FN fibers. With regard to FN injuries due to cranial trauma or inflammation, the anatomical structure of the nerve is often conserved. Therefore, the incidence of incomplete facial paralysis is relatively higher clinically. In those cases, surgical intervention is still desirable for patients with significant incomplete facial paralysis, such as with House–Brackmann (H-B) grade IV or V on FN function (1). However, this issue raises the question of how to address the injured FN when using hypoglossal–facial nerve neurorrhaphy (HN-FN) or other nerve transfer methods. In previous studies, we modified the classical method of HN-FN side-to-end neurorrhaphy by side-to-side to preserve the remaining FN fibers and the possibility of spontaneous regrowth of the FN, showing that innervations of both HN and FN fibers could be achieved with the return of certain facial functions (2, 3). However, the importance and usefulness of the remaining or few spontaneously regenerated FN fibers in regaining facial function when using HN-FN neurorrhaphy to treat facial paralysis remain controversial. Some authors considered that the injured FN could be completely sacrificed for HN-FN neurorrhaphy if facial paralysis needs to be surgically treated even if a few regenerated or remaining FN fibers existed, stressing that regenerated HN fibers alone could effectively induce facial reanimation (4, 5). However, several other studies have mentioned the importance of the remaining or regenerated FN fibers (6–9).

To clarify these questions, we designed a rat FN injury model with preservation of its anatomical structure followed by HN-FN side-to-side neurorrhaphy to investigate the effects of the remaining and/or spontaneously regenerated FN fibers on reestablished facial function. After evident facial function returned following FN injury and neurorrhaphy, the FN was cross-sectioned according to different ratios at the site rostral to the initial FN injury to retain, partially abolish, or completely abolish the spontaneously regenerated FN fibers that had successfully regrown through the injury site. Then, facial function was assessed using clinical observation and electrophysiological examination, as well as histological evaluation. We hope that this study provides useful information for neurosurgeons to determine the method of HN-FN neurorrhaphy for treating anatomical structure-preserved FN injuries.

## Materials and Methods

Forty-five adult male Sprague–Dawley (SD) rats (weighing 180–200 g; Beijing Vital River Laboratory Animal Technology Co., Ltd, Beijing, China) were used in this study. All experiments were approved by the Local Animal Ethics Committee with reference number SYXK2019-0007.

Rats were divided into the sham control group (*n* = 5) and experimental group (*n* = 40). In the experimental group, FN injury followed by HN-FN side-to-side neurorrhaphy was first performed in all rats using a predegenerated nerve graft (PNG). Three months later, experimental rats were further divided into four subgroups: retaining the tissue structure of the injured FN (Group E1, *n* = 10), cross-sectioning one-third of the FN at the site rostral to the initial FN injury (E2, *n* = 10), cross-sectioning two-thirds of the FN (E3, *n* = 10), or complete cross-sectioning of the FN (E4, *n* = 10).

### FN Injury and HN-FN Side-to-Side Neurorrhaphy

All experimental rats underwent general anesthesia with intraperitoneal injection of sodium pentobarbital (54 mg/kg). The right FN was exposed from the stylomastoid foramen to its bifurcation site under a neurosurgical microscope (Leica M650). Muscle action potentials (MAPs) were recorded at the right whisker pad, while the exposed FN trunk was electrostimulated using an electrophysiological stimulator (EB-Neuro). The FN was then crushed using micro forceps for 1 min under the neurosurgical microscope, certainly sectioning all contained axons with a gap of 3 mm except for the epineurium. In order to standardize the injury extent and furthermore limit the spontaneous regrowth of the sectioned facial axons within the rats, the crush site was ligated by 4-0 nylon sutures. Under this condition, ~30% of rat FN axons could regenerate through the injury site into the distal FN segment (10). Complete section of the FN was not performed while making the injury because our objective was to induce a limited spontaneous regrowth of injured FN axons through the injury site into the distal FN segment. Complete section of the FN followed by neurorrhaphy between the two nerve stumps was likely to just increase complexity of the surgical operations.

**Figure 1:**
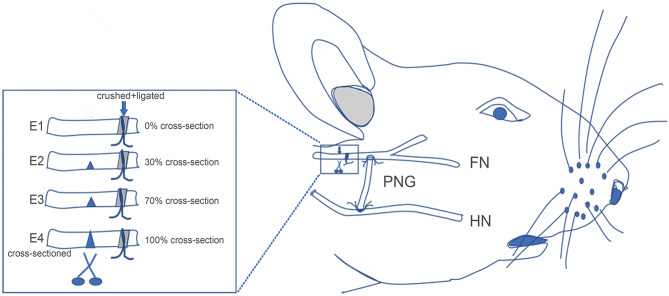
Schematic showing the surgical procedures for facial nerve (FN) injury, hypoglossal–facial nerve (HN-FN) side-to-side neurorrhaphy, and spontaneously regenerated FN sectioning. **(Right)** After the exposure of the FN and HN, the FN crush injury and HN-FN neurorrhaphy with a predegenerated nerve autograft were performed. **(Left)** Three months after the neurorrhaphy and return of facial function. FN sectioning was performed rostral to the ligation site.

HN-FN side-to-side neurorrhaphy was performed through an interposed PNG (Figure 1). At the FN trunk distal to the injury site, an epineural window of ~5-mm diameter was created using micro scissors. The HN was exposed under the digastric muscle. A PNG was prepared 1 week before by crushing and ligating the right sural nerve of the same rat, as described in previous studies (10, 11). A 10-mm-long PNG was removed. One end of the PNG was surgically bridged using 10-0 nylon sutures end-to-side to the FN at the epineural window, and another end was sutured to the exposed HN at the site where the nerve was cross-sectioned by 50%.

### Sectioning of the Regenerated FN

After 3 months of follow-up, experimental rats were reanesthetized as in the initial surgery. The FN was carefully re-exposed, and the injury/ligation site was identified. The FN immediately rostral to the injury/ligation site was cross-sectioned according to different ratios of the width of the FN trunk (Figure 1). In subgroup E1, no cross-sectioning was performed, and the regenerated FN was retained (0%). In subgroup E2, the FN was cross-sectioned by one-third (~30%). In subgroup E3, the FN was cross-sectioned by two-thirds (~70%). In subgroup E4, the FN was completely cross-sectioned (100%). One week later, the rats were evaluated with facial function assessment, electrophysiology, and histology.

In sham control rats, the FN and HN were exposed only with no injury and neurorrhaphy.

### Facial Symmetry at Rest

Each rat was regularly photographed at rest before and after surgery. The angle α was measured and recorded (10, 12). Briefly, the angle α was measured between a line extending from the fold on the bridge of the nose and a line linking the outer corners of the eyes.

### Eye Blinking Activity

The blinking reflex indicates the integrity of the reflection arc from the trigeminal afferent to the FN efferent (13, 14). Eye blinking activity was induced in rats by blowing air on their eyes using an aurilave and recorded with a camera before and after surgery. A standardized facial grading system was used to evaluate this activity [Table 1; (15)].

**Table 1:** Classification of eye blinking activity.

**Eye blinking activity**	
1	Absence of eye blinking reflex and closure
2	Presence of orbicular muscle contraction, without the blinking reflex
3	50% eye closure through the blinking reflex
4	75% closure through the blinking reflex
5	Presence of complete eye closure and the blinking reflex

### Vibrissae Movement Analysis

The video-based assessment of vibrissae motion was performed as previously described to evaluate dynamic facial function (16, 17). Under anesthesia with isoflurane, all rat vibrissae were clipped except for the two large whiskers in row C on both sides. Vibrissae movements of protraction and retraction were video recorded during rat exploration. A Quintic Biomechanics (v26) was used for the analysis, and the biometric model was reformed according to Tomov's method (17). The analysis index included: (1) amplitude (the angle between protraction and retraction); (2) angular velocity (degrees per second between protraction and retraction); (3) angular acceleration (degrees per second square between protraction and retraction).

### Synkinesis Observation

Synkinesis is involuntary movement of the facial muscles associated with voluntary facial movement (18). We videotaped the eyelid movement in rats during eating. For analysis, the two points at the intersections of the upper and lower eyelids through the vertical line of the pupil were marked with a biometric method. The analysis index was the ratio of the shortest distance between the two points during eating to the distance when the eyes were opening without eating.

### Electrophysiological Examination

We recorded MAPs using electromyography at the right whisker pad in response to stimulation of either the injured FN at the site rostral to the neurorrhaphy or the PNG trunk at the middle point (10, 19–21). Stimulation (0.2 ms, 2 mA) was delivered through two 0.5-mm-diameter electrodes directly placed onto the FN and PNG trunk. The MAPs were recorded by another two 0.5-mm-diameter electrodes inserted into the whisker pad. The amplitudes between the largest positive and negative peaks and the surface under the amplitude were measured.

### Fluorescent Retrograde Labeling Regenerated Motoneurons

Fluorescent tracers were injected into the paralyzed/reinnervated facial muscle to retrogradely detect the regenerated motoneurons in the facial and hypoglossal nuclei. Rats were anesthetized as in the initial surgery, and 10 μl of 2.5% Fluoro-Gold (FG; Sigma) was injected into the right orbicularis at multiple points, while 20 μl of 1% cholera toxin subunit B conjugated to Alexa Fluor 555 (CTB-Alexa 555; ThermoFisher) was injected into the right whisker pad. One week later, rats were killed by intraperitoneal overdose injection of pentobarbital (120 mg/kg). Intracardiac perfusion with 300 ml of phosphate-buffered saline (0.1 M, pH 7.2) followed by 200 ml of 4% paraformaldehyde (PFA) was then performed. The brainstem was removed and preserved in 4% PFA solution for 3 h. After gradient dehydration in sucrose solutions, specimens were immersed in optimal cutting temperature (OCT; Sakura, USA) compound and cross-sectioned at a thickness of 25 μm using a freezing microtome (CM1950; Leica, Germany). Fluorescently labeled motoneurons were identified and counted using a Zeiss Axio Imager 2 imaging optic fluorescence microscope (Axio Imager M2, Zeiss, Germany).

### Axonal Counting in the Reconstructed Nerve Pathway

The middle point of the PNG and the FN trunk immediately caudal to the initial injury site but rostral to the PNG-FN neurorrhaphy site were removed after perfusion with 4% PFA and preserved in 3.6% glutaraldehyde for 3 h. Specimens were post-fixed with osmium tetroxide and immersed in Epon. Semithin cross-sections (0.35 μm) were acquired using an ultramicrotome (Reichert Ultracut S Wild M3z, Leica) and observed under the optic microscope. Myelin was quantified using ImageJ 1.47.

### Statistical Analysis

All data are presented as the means ± SD. Statistical analysis was performed using SPSS 23.0 (IBM), and graphs were drafted using GraphPad Prism 7 (GraphPad Software). Group differences were analyzed by a Kruskal–Wallis test or one-way ANOVA followed by LSD, Bonferroni, or Tamhane *post-hoc* tests. Values of *P* < 0.05 were considered statistically significant (^***^P < 0.001 or ###*P* < 0.001; ^**^P < 0.01 or ^##^*P* < 0.01; ^*^P < 0.05 or ^#^*P* < 0.05).

## Results

### Facial Symmetry at Rest

**Figure 2:**
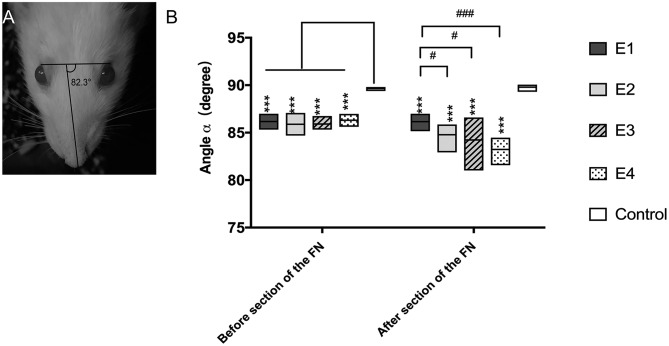
**(A)** The angle α was measured between a line extending from the fold on the bridge of the nose and a line linking the outer corners of the eyes, and the measurement was performed three times per rat. **(B)** Measurement of the angle α 3 months after the neurorrhaphy and 1 week after FN sectioning. The results are presented as the means ± SD. The difference among the groups was analyzed by one-way ANOVA. Compared with the experimental groups before or after FN sectioning, the control group had significantly higher values with the measurement (***P = 0.000). Before FN sectioning, there was no difference among the four experimental groups (E1 to E4). After FN cross-sectioning, for E1 vs. E2, ^#^*p* = 0.021; E1 vs. E3, ^#^*p* = 0.043; E1 vs. E4, ^###^*p* = 0.000.

When rats were at rest, the angle α was 89.65 ± 0.16° in intact rats, but it decreased to 84.39 ± 0.53° when the FN was injured and immediately repaired by HN-FN side-to-side neurorrhaphy. Three months after neurorrhaphy, the angle α reached 86.07 ± 0.2° in experimental rats. One week after FN sectioning according to different ratios, the angle α of all experimental rats decreased except that of those in subgroup E1 without FN sectioning. In subgroup E2, the α decreased to 84.78 ± 0.90°. In subgroup E3, the α decreased to 84.23 ± 1.61°, while the α decreased to 83.22 ± 0.98° in subgroup E4. Statistical analysis revealed significant differences between subgroup E1 and other subgroups (Figure 2).

### Eye Blink Activity

**Figure 3:**
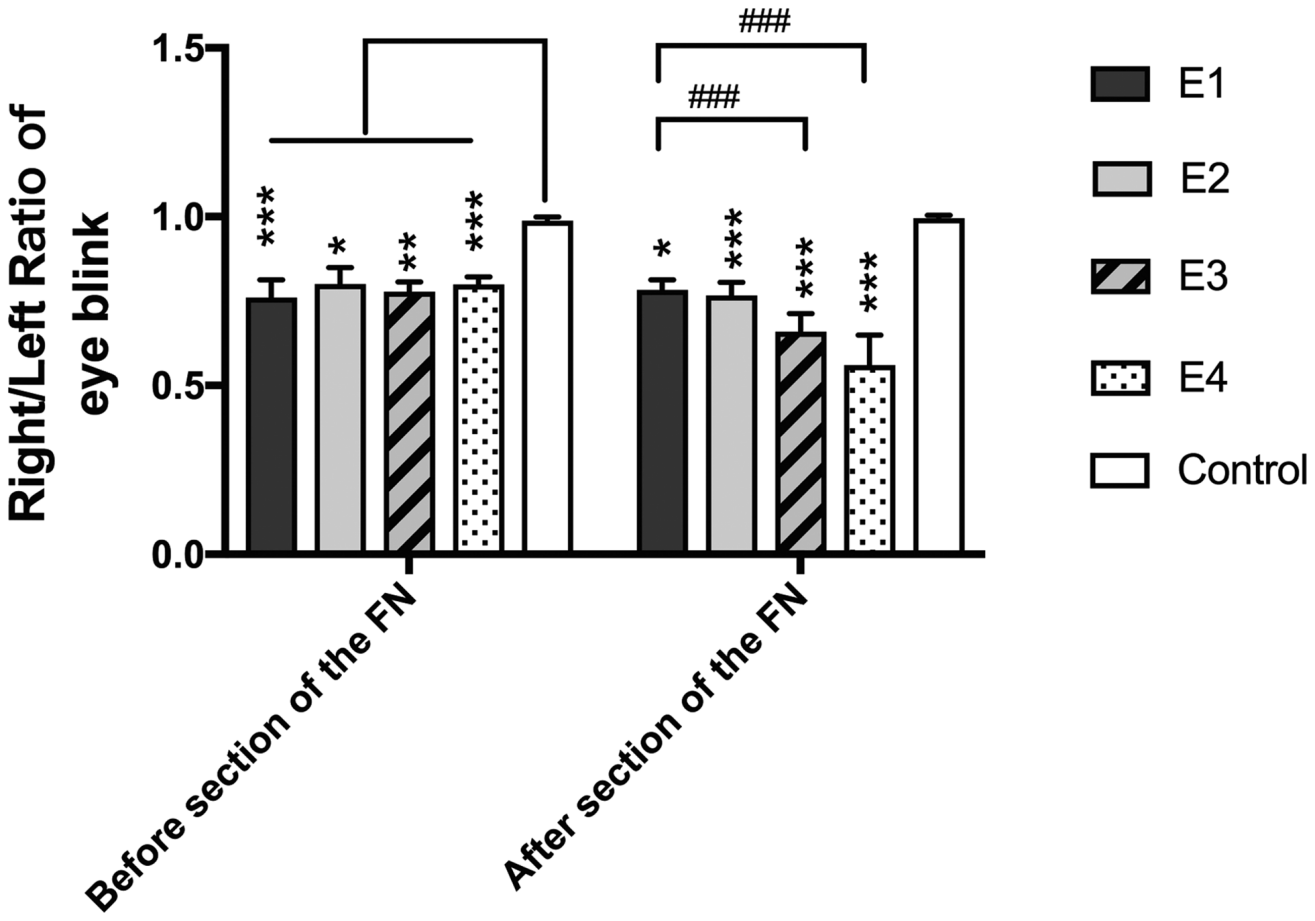
The right to left ratio of the eye blinking score was measured. All the values are shown as the means ± SD and analyzed by the Kruskal–Wallis test. Three months after neurorrhaphy before FN sectioning, the significant difference was evident between any experimental group and the control group (E1 vs. Control, ***P = 0.000; E2 vs. Control, *P = 0.02; E3 vs. Control, **P = 0.003; E4 vs. Control, ***P = 0.000), although there was no difference among the four experimental groups. One week after FN cross-sectioning with different ratios, subgroup E3 (^###^*p* = 0.000) and E4 (^###^*p* = 0.000) became much worse than E1, and the difference between the control group and the experimental group was obvious (E1 vs. Control,*P = 0.01; E2 vs. Control, ***P = 0.001; E3 vs. Control, ***P = 0.001; E4 vs. Control, ***P = 0.001).

In intact rats, the eye blinking score was 4.94 ± 0.05. In experimental rats, the recorded score was 3.77 ± 0.25 in subgroup E1, 3.98 ± 0.25 in E2, 3.88 ± 0.12 in E3, and 3.97 ± 0.11 in E4 3 months after neurorrhaphy. When the FN was sectioned according to different ratios, the scores decreased to 3.83 ± 0.2 in E2, 3.29 ± 0.27 in E3, and 2.8 ± 0.44 in E4, but no evident change was observed in E1 without FN sectioning (Figure 3). E1 was significantly different from E3 and E4. Although no significant difference was found between E1 and E2, a tendency toward eye blinking reduction was shown.

### Vibrissae Movement Analysis

**Figure 4:**
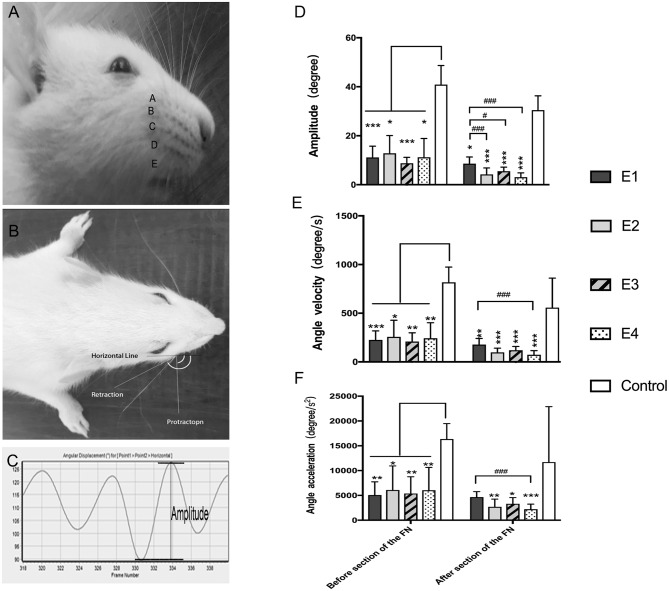
**(A)** The rat's vibrissae were arranged into five rows, and the largest whisker in row C was selected. **(B)** Photograph showing the angle between the protraction and the retraction recorded between the horizontal line and the whisker extension line defined between the root of the whisker and one-eye distance away from the root, which represent the degree of the amplitude of whisker movement. **(C)** The graphs recorded by Quintic Biomechanics showing the amplitude. The amplitude value was measured between the peaks in one cycle (the angle between protraction and retraction), angular velocity was calculated by the recorded amplitude per second, and angular acceleration was measured by the amplitude per second square. **(D)** The recorded amplitude values were presented as the means ± SD and analyzed by the Kruskal–Wallis test and LSD *post-hoc* test. The amplitude of the control group was higher than any other test groups regardless of FN sectioning. Before FN sectioning, E1 vs. Control, ***P = 0.000; E2 vs. Control, *P = 0.04; E3 vs. Control, ***P = 0.000; E4 vs. Control, *P = 0.02. One week after FN sectioning E1 vs. Control, *P = 0.01; E2 vs. Control, ***P = 0.000; E3 vs. Control, ***P = 0.000; E4 vs. Control, ***P = 0.000). Before the cross-sectioning procedure, the four experimental groups had no difference; E1 became higher than E2 (^###^*p* = 0.000), E3 (^#^*p* = 0.02), and especially E4 (^###^*p* = 0.000) after FN cross-sectioning according to different ratios. **(E)** The measurement of angle velocity is presented as the mean ± SD. The difference was calculated with the Kruskal–Wallis test and LSD *post-hoc* test. Before FN cross-sectioning, the four experimental groups showed no difference and were significantly lower than the control group (E1 vs. Control, ***P = 0.000; E2 vs. Control, *P = 0.02; E3 vs. Control, **P = 0.004; E4 vs. Control, **P = 0.007). After FN section, although the means of each group decreased, the mean of the control group remained higher than that of any other experimental group (E1 vs. Control, **P = 0.01; E2 vs. Control, ***P < 0.000; E3 vs. Control, ***P = 0.000; E4 vs. Control, ***P = 0.000), and compared with the E1 group, the E4 group (^###^*p* = 0.000) was especially decreased. (**F**) Means ± SD are shown to analyze the angle acceleration before FN sectioning. The difference among the groups was consistent with the amplitude (E1 vs. Control, **P = 0.001; E2 vs. Control, *P = 0.04; E3 vs. Control, **P = 0.01; E4 vs. Control, **P = 0.01). After FN cross-sectioning, though the means decreased in the five groups, the trend of decreased angle acceleration in the experimental groups was obvious; ^#^*p* = 0.001, for the E1 group vs. E4 and the experimental groups showed significant low value when compared with the control group (E2 vs. Control, **P = 0.004; E3 vs. Control, *P = 0.02; E4 vs. Control, **P = 0.000).

In intact rats, the vibrissae presented an anterior position with an amplitude of 38.55 ± 7.56°, an angle velocity of 818.1 ± 155.34°/s, and an angle acceleration of 16,362 ± 3,106.72°/s^2^. Immediately after FN injury and neurorrhaphy, the vibrissae movement was paralyzed on the affected side of the face. Three months later, the vibrissae amplitude recovered to 11.16 ± 4.56° in subgroup E1, 12.83 ± 7.24°in E2, 8.74 ± 2.45° in E3, and 11.21 ± 7.65° in E4. The angle velocity recovered to 225.31 ± 93.35°/s in E1, 256.75 ± 171.18°/s in E2, 208.72 ± 90.21°/s in E3, and 242.49 ± 159.48°/s in E4. The angle acceleration reached 5,078.14 ± 2,666.25°/s^2^ in E1, 6,089.59 ± 4,859.59°/s^2^ in E2, 5,382.49 ± 3,391.21°/s^2^ in E3, and 6,035.01 ± 4,617.14°/s^2^ in E4. After FN sectioning according to different ratios, the vibrissae amplitude decreased again to 4.23 ± 2.58° in E2, 5.54 ± 1.62° in E3, and 3.09 ± 1.72° in E4; only a slight decrease to 8.58 ± 2.76° was observed in E1 with no FN sectioning. The angle velocity decreased to 178.61 ± 62.38°/s in E1, 98.79 ± 41.62°/s in E2, 138.05 ± 47.08°/s in E3, and 73.46 ± 42.5°/s in E4. The angle acceleration decreased to 4,670.86 ± 1,102.07°/s^2^ in E1, 2,701.62 ± 1,554.43°/s^2^ in E2, 3,317.54 ± 1,253.33°/s^2^ in E3, and 2,217.3 ± 1,027.9°/s^2^ in E4 (Figure 4).

### Synkinesis Observation

Synkinesis was observed in all experimental rats during eating after FN injury and neurorrhaphy. After FN sectioning according to different ratios, no significant difference of synkinesis was observed between the experimental subgroups.

### Electrophysiological Examination

Three months after HN-FN neurorrhaphy, MAPs were recorded at the whisker pad in response to electrostimulation of the FN or PNG. The amplitude was 2.75 ± 0.45 mV, and the surface was 6.36 ± 3.86 mVms during electrostimulation of the FN in experimental rats. When the PNG was electrostimulated at the middle point, MAP values of 1.89 ± 0.96 mV for the amplitude and 3.71 ± 2.74 mVms for the surface were recorded.

**Figure 5:**
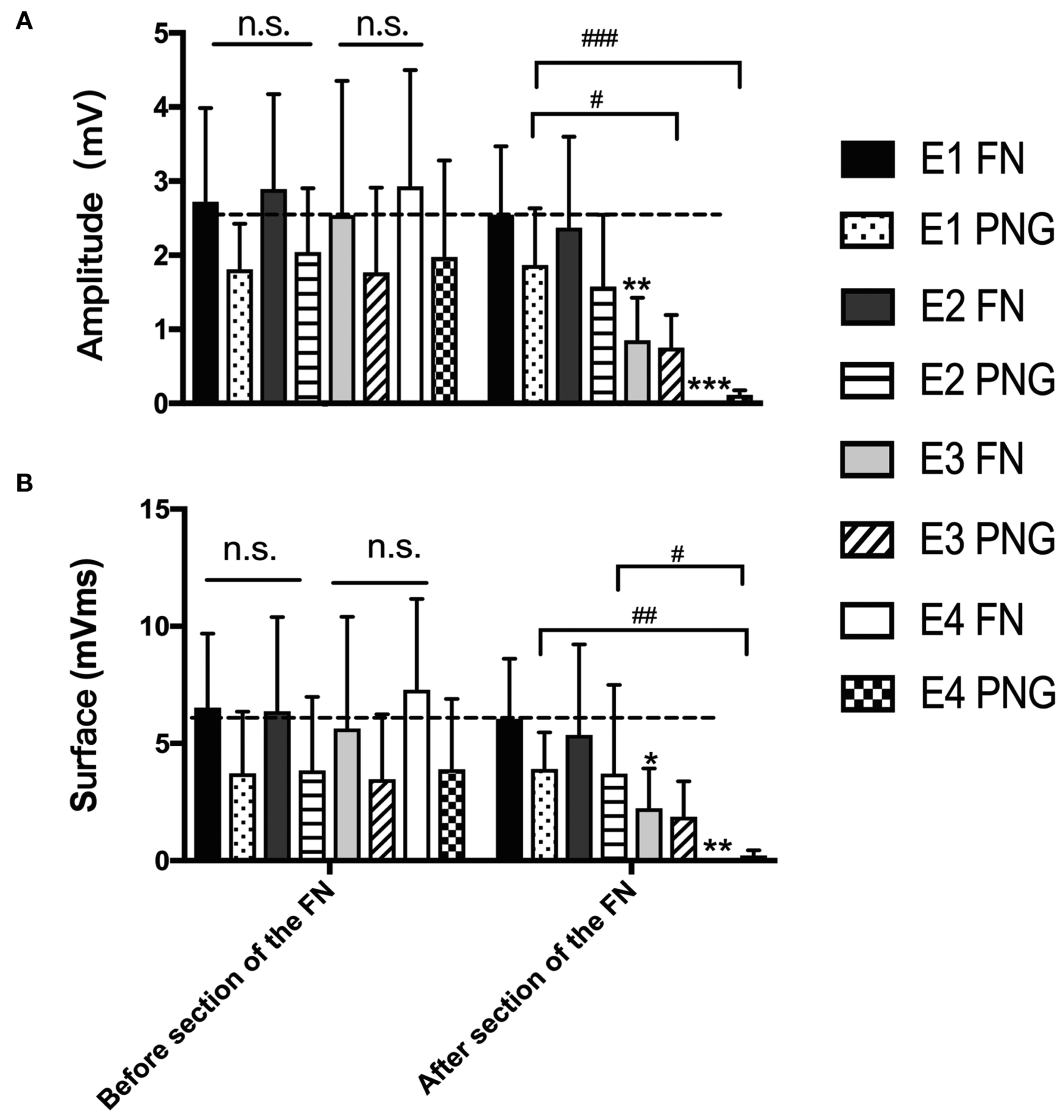
**(A)** The recorded MAP amplitudes at the whisker pad when the FN and PNG were electrostimulated are presented as the means ± SD. The statistics were analyzed by one-way ANOVA and the Tamhane *post-hoc* test. Regardless of FN sectioning, the amplitude was higher when stimulating the FN than when stimulating the PNG. Before FN sectioning, there was no difference (n.s.) among the four experimental groups when the FN or PNG was stimulated. After FN sectioning, and stimulating the FN, **p = 0.003, for E3 compared to groups with mean values at the dotted line, which indicated group E1, ***p = 0.000, for E4 compared to E1; in addition, there was a significant difference between E2 (*p = 0.019) and E4 and between E3 (*p = 0.025) and E4. When stimulating the PNG, the tendency of the difference was consistent with that of the FN. ^#^*p* = 0.015, for the amplitude of E3 vs. that of E1; ^###^*p* = 0.001, for the amplitude of E4 vs. that of E1. **(B)** The results of MAP surface recorded and calculated at the whisker pad when the FN and PNG were stimulated are presented as the means ± SD. The trend was the same as that of the amplitude. The statistics were analyzed by one-way ANOVA. Regardless of FN sectioning, the surface was higher when stimulating the FN than the PNG. Before sectioning of the FN, there was no difference (n.s.) among the four experimental groups regardless of whether the FN or PNG was stimulated. After FN cross-sectioning, and stimulating the FN, *p = 0.042, for E3 compared to groups with mean values at the dotted line, which indicated group E1, and **p = 0.005, for E4 compared to E1. When stimulating the PNG, the tendency of the difference was consistent with that of the FN. ^##^*p* = 0.006, for the surface of E4 vs. that of E1; ^#^*p* = 0.013, for the surface of E4 vs. that of E2.

One week after FN sectioning according to different ratios, the recorded MAPs in response to electrostimulation of the FN were 2.55 ± 0.92 mV for the amplitude and 6.05 ± 2.567 mVms for the surface in E1, 1.87 ± 1.21 mV and 5.37 ± 3.86 mVms in E2, 0.85 ± 0.57 mV and 2.24 ± 1.70 mVms in E3, and 0.002 ± 0.007 mV and 0.009 ± 0.27 mVms in E4, respectively. When electrostimulating the PNG, the recorded values were 1.87 ± 0.77 mV for the amplitude and 3.91 ± 1.56 mVms for the surface in E1, 1.58 ± 0.97 mV and 3.70 ± 3.79 mVms in E2, 0.75 ± 0.44 mV and 1.88 ± 1.51 mVms in E3, and 0.12 ± 0.61 mV and 0.21 ± 0.22 mVms in E4 (Figure 5).

### Fluorescent Retrograde Labeling of Regenerated Motoneurons

**Figure 6:**
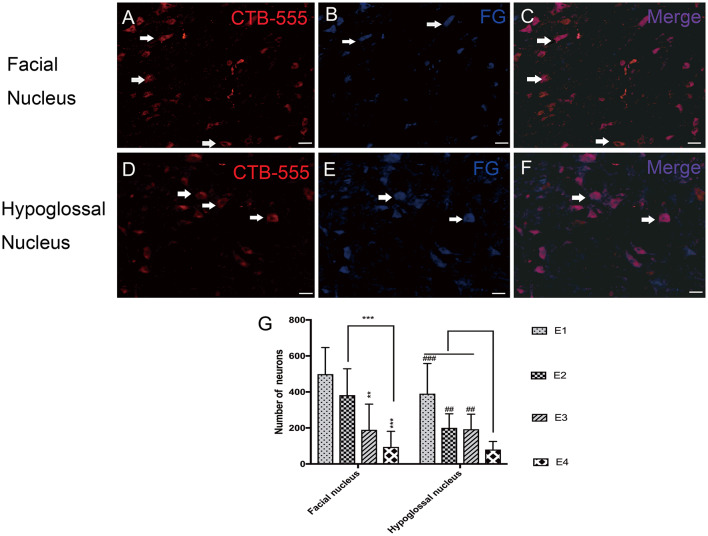
**(A–C)** Retrograde labeled neurons in the facial nucleus. **(A)** CTB-555-labeled neurons (arrows) in the facial nucleus. **(B)** FG-labeled neurons (arrows) in the facial nucleus. **(C)** Double-labeled neurons (arrows) in the facial nucleus. **(D–F)** Retrograde labeled neurons in the hypoglossal nucleus. **(D)** CTB-555-labeled neurons (arrows) in the hypoglossal nucleus. **(E)** FG-labeled neurons (arrows) in the hypoglossal nucleus. **(F)** Double-labeled neurons (arrows) in the hypoglossal nucleus. Scale bars: 50 μm. **(G)** Retrograde-labeled neurons were quantified 1 week after FN sectioning. The data are presented as the means ± SD, and differences were tested by the Wallis–Kruskal test. In the facial nucleus, the number of total labeled neurons was decreased, consistent with FN cross-sectioning. **p = 0.002, for the neurons in E3 vs. E1 and ***p = 0.000, for the neurons in E4 vs. E1, ***p = 0.000, for the neurons in E4 vs. E2. In the hypoglossal nucleus, the trend is very similar; the number of neurons in E1 is significantly higher than that in E4 (^###^*p* = 0.000), and E2 contains more neurons than E4 (^##^*p* = 0.002). The number of neurons in E3 is higher than that in E4 (^##^*p* = 0.008).

In the facial nucleus, 497 ± 148 labeled neurons were counted in subgroup E1, 382 ± 146 in E2, 190 ± 143 in E3, and 94 ± 87 in E4, respectively. In the hypoglossal nucleus, 391 ± 168 neurons were counted in subgroup E1, 201 ± 78 in E2, 193 ± 83 in E3, and 80 ± 45 in E4 (Figure 6).

### Axonal Counting in the Reconstructed Nerve Pathway

**Figure 7:**
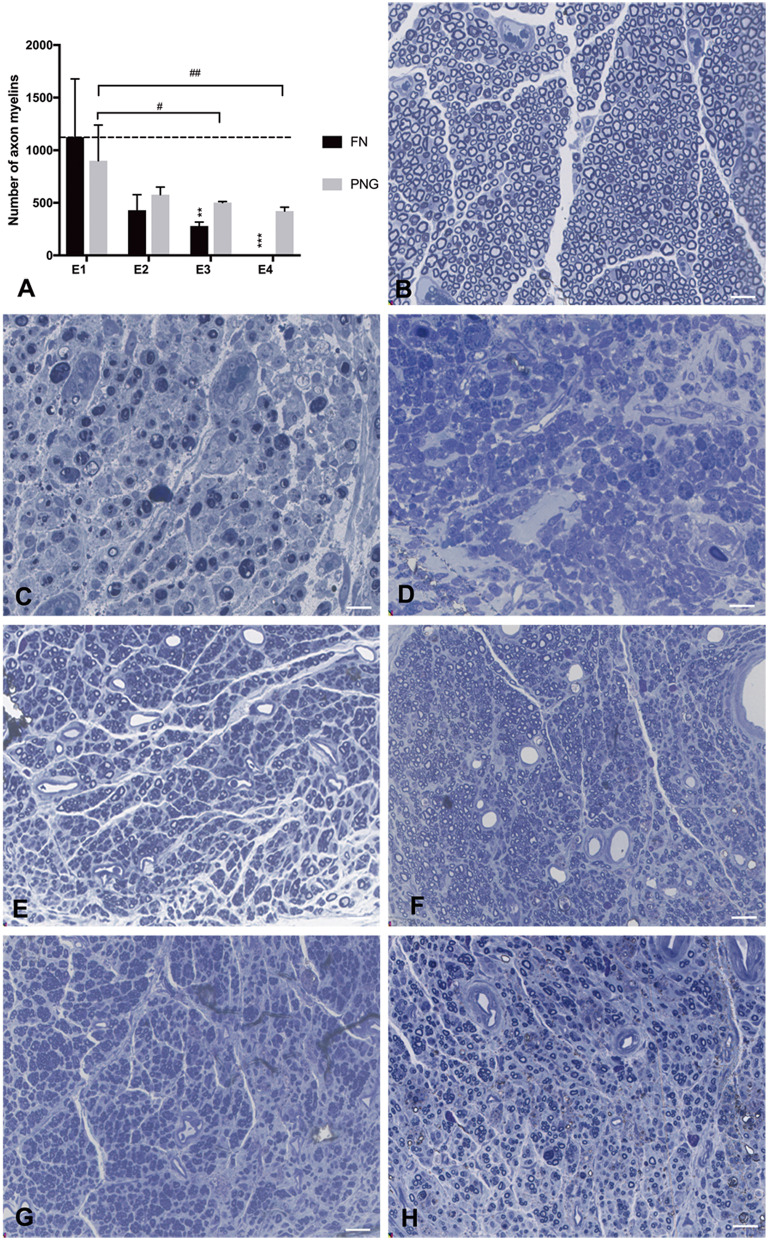
Axonal counting was performed by scoring the number of myelinated axons in the semithin sections of the FN at the level of FN trunk immediately caudal to the initial injury site but rostral to the PNG–FN neurorrhaphy site or the PNG at the middle point level. **(A)** The number of myelinated axons of the FN and PNG in the four experimental groups was calculated 1 week after the sectioning and is presented as the mean ± SD. The difference was analyzed with one-way ANOVA and the Bonferroni *post-hoc* test. For the FN, **p = 0.007, the number of myelinated axons in E3 compared to the group with a mean value at or below the dotted line or as indicated in E1. ***p = 0.001, E4 compared with E1. For the PNG of the four groups, E3 (^#^*p* = 0.032) and E4 (^##^*p* = 0.01) contained significantly less axonal myelin than did E1. **(B)** The section of the normal FN trunk with abundant axonal myelin. **(C)** The section of the 7-day injured FN trunk with massive axonal degeneration. **(D)** The sectioning of the FN trunk 1 week after the FN sectioning in E4, similar to **(C)**, massive axonal degeneration. **(E–H)** The axonal myelin of the PNG at the middle point level for E1 to E4. Scale bars: 300 μm.

There were 1,125 ± 553 myelinated axons counted in E1, 429 ± 150 in E2, 280 ± 37 in E3, and 0 in E4 at the level of the FN trunk immediately caudal to the initial injury site but rostral to the PNG–FN neurorrhaphy site after FN sectioning according to different ratios. In the PNG, there were 901 ± 339 myelinated axons counted in E1, 578 ± 73 in E2, 502 ± 10 in E3, and 422 ± 37 in E4 (Figure 7) at the middle point level.

## Discussion

Satisfactory facial reanimation depends on the return of facial symmetry at both rest and action. Among these facial symmetries, restoration of the symmetry of facial action is more challenging. The return of muscle contraction force, which is even more powerful, has been achieved using HN transfer to the FN with end-to-end or side-to-end neurorrhaphy (6, 22–24). However, recovery of the symmetry of facial action remains unsatisfactory in most cases, probably because of ectopic innervation of paralyzed facial muscles by hypoglossal motoneurons. Although the shared innervation of the facial and hypoglossal nuclei exists within the brainstem, and intense facial exercise is likely to promote central plasticity and facilitate the development of new functions for hypoglossal motoneurons to improve facial function (25, 26), the extent of central plasticity is probably limited, and the real compensation of facial function is weaker. Notably, resting facial symmetry and tone as well as powerful movements could be recovered by the innervation of hypoglossal motoneurons, and more physiological recovery of facial function may be induced by FN reinnervation (27), indicating the importance of participation of FN axons in regaining facial function after HN-FN neurorrhaphy. In previous studies, HN-FN side-to-side neurorrhaphy preserved the remaining and/or spontaneously regenerating injured facial axons while inducing innervation of paralyzed facial muscles by HN fibers, leading to facial and hypoglossal double axonal innervation (2, 3). In these patients, conversion of hypoglossal function to facial function occurred in most cases post-operatively. For example, voluntary facial movement without tongue contraction or the intention of tongue contraction was observed several months following neurorrhaphy, unlike the movement associated with tongue contraction in the early post-operative period. In this study, we further investigated the role of remaining and/or spontaneously regenerated facial axons in regaining facial function; these axons played an important role in axonal innervation as well as in facial function restoration. Once these axons were sectioned, the innervation and restored function were quantitatively and qualitatively lost according to the sectioning ratio of the FN.

Interestingly, in this study, the remaining and/or regenerated facial axons also played a role in innervation of paralyzed facial muscles by hypoglossal motoneurons, which was most likely consistent with the “babysitter” concept (7, 27, 28). Furthermore, these facial axons had an impact on the regenerated hypoglossal motoneurons that had innervated the facial muscle, such as the reduction in the hypoglossal motoneurons that occurred in a quantitative manner when facial axons were later sectioned according to different ratios.

Reestablishment of functional connections between the motor and sensory nervous systems is also crucial for functional recovery after nerve repair. Regarding facial function, blinking activity indicates the integrity of the reflection arc from the trigeminal afferent to the FN efferent through internal links between the facial and trigeminal nuclei. In this study, blinking activity could be recovered following HN-FN neurorrhaphy, but additional damage induced by later facial nerve sectioning resulted in a decrease in this activity that quantitatively corresponded to the sectioning ratio, indicating the importance of these remaining and/or spontaneously regenerated facial axons.

Synkinesis is a major, important complication following neurorrhaphy, particularly after ectopic neurorrhaphy. The incidence of synkinesis can be reduced after facial rehabilitation surgery if the facial muscles are innervated not only by transposed hypoglossal axons but also by the remaining FN axons because the shared innervation of the facial and hypoglossal nuclei within the brainstem may prevent synkinesis of the doubly innervated facial muscles (29–32). In this study, synkinesis was observed in all experimental rats following FN injury and HN-FN neurorrhaphy. However, no significant difference was established between the subgroups after FN sectioning according to different ratios. This lack of a difference was likely due to our gross observation and short post-operative follow-up period.

## Conclusion

This study shows that the remaining and/or spontaneously regenerated facial axons played an important role in innervation of paralyzed facial muscles and restoration of function when using HN-FN neurorrhaphy to treat facial paralysis due to FN injury. Combined with the participation of these facial axons, post-operative rehabilitation exercises may largely promote facial functional recovery.

## Data Availability Statement

The raw data supporting the conclusions of this article will be made available by the authors, without undue reservation, to any qualified researcher.

## Ethics Statement

This animal study was reviewed and approved by Local Animal Ethics Committee (SYXK2019-0007).

## Author Contributions

This study was conducted by YZ. ML and ZL gave analysis support. DL, HW, and MS gave directions for the experiment. SL gave the directions for all the study.

## Conflict of Interest

The authors declare that the research was conducted in the absence of any commercial or financial relationships that could be construed as a potential conflict of interest.
